# An explainable artificial intelligence framework for ischemic heart disease prediction using enhanced squirrel search feature selection

**DOI:** 10.1038/s41598-026-46823-0

**Published:** 2026-04-01

**Authors:** D. Cenitta, N. Arul, R. Vijaya Arjunan, Krishnaraj Chadaga, J. Andrew

**Affiliations:** 1 Manipal Institute of Technology, Manipal Academy of Higher Education, Manipal, India; 2 Department of Computer Science & Engineering, AJ Institute of Engineering and Technology, Mangalore, 575006 Karnataka India

**Keywords:** Ischemic heart disease (IHD), Explainable artificial intelligence (XAI), Squirrel search optimization (SSO), SHAP, LIME, Feature selection, Random forest, Medical diagnosis, Machine learning, Model interpretability, Good health and well being, Computational biology and bioinformatics, Health care, Mathematics and computing

## Abstract

Clinical decision-making depends on the ability to anticipate ischemic heart disease (IHD) accurately and interpretably. Despite their excellent accuracy, machine learning models are not often accepted in medical applications due to their black-box nature. To diagnose IHD, this study suggests an Explainable Artificial Intelligence (XAI) architecture that combines explainable models with the Enhanced Squirrel Search Optimization (ESSO) method for feature selection. The proposed Enhanced Squirrel Search Optimization (ESSO) introduces adaptive exploration mechanisms to efficiently identify an optimal subset of clinically relevant features. By integrating ESSO-based feature selection with Random Forest classification and explainable AI techniques (SHAP and LIME), the framework simultaneously improves predictive performance and provides interpretable insights for clinical decision support. We use SHAP (SHapley Additive exPlanations) and LIME (Local Interpretable Model-Agnostic Explanations) methodologies to offer clear explanations into the model’s predictions, building on earlier research that showed how effective squirrel search is at selecting the best clinical features. With KNN-based imputation, normalization, categorical encoding, outlier handling, and SMOTE-based class balancing for managing missing data, the UCI heart disease dataset is utilized for both training and validation. Random Forest is used to classify the chosen features, and domain experts assess the derived explanations for clinical relevance. With precise visual and numeric explanations of contributing features including cp., oldpeak, thal, and ca., the suggested model achieves an accuracy of 98.4%. By bridging the gap between interpretability and high-performance machine learning, our research makes the model appropriate for practical use in clinical settings.

## Introduction

One of the main causes of death in the world today is Ischemic Heart Disease (IHD), also referred to as coronary artery disease. According to estimates from the World Health Organization (WHO), cardiovascular diseases (CVDs) accounted for 17.9 million deaths globally in 2016—nearly 31% of all deaths^1^. Ischemic heart disease accounted for a significant portion of these deaths, particularly in low- and middle-income countries. The diagnosis of IHD has become a clinically important task since the global population is aging and remains under threat of risks linked to lifestyle including smoking, obesity, diabetes, and hypertension^2^.

With the increased popularity of enhancements to diagnostics, artificial intelligence (AI) and machine learning (ML) methods have sequentially become a part of healthcare systems to facilitate IHD diagnosis and prognosis. These techniques using data have been found to be highly effective at analyzing relationships and patterns in medical data not discernible through conventional clinical assessment^3,4^. With the past records of the patients, the machine algorithms of machine learning can predict the probability of cardiac sickness with much accuracy. Yet, the quality and the relevance of training features play an important part in the successfulness of working of these models. Redundancy or repeating features are common in high dimensional datasets that are usually used in IHD studies, which has the effect of obscuring predictive signals, leading to overfitting and increasing computing costs^5^.

To solve this problem, approaches to feature selection are used to select and retain the most information-rich features of a dataset. Due to their ability to exploit comprehensive solution spaces as well as their resemblance to the natural processes, metaheuristic algorithms have become popular in issues of feature selection. Among them, the Squirrel Search Algorithm (SSA), introduced in 2019 stands out since it balances its exploration and exploitation of searching spaces, and is informed by a similar phenomenon observed to happen in nature: the active foraging process of flying squirrels^6^. The system imitates gliding movements and seasonal movement of squirrels in deciduous forests to obtain the best answers. To enhance the convergence velocity and accuracy, better mobilizations of SSA such as the Improved Squirrel Search Optimization (ISSO) make other enhancements such as the use of an adaptive probability of the presences of a predator and the jumping-gliding mechanisms^7^.

Recent studies have revealed that SSA and its variants do comparatively well in feature selection in many areas of science including the identification of heart disease. To take an example, Cenitta et al.^8^ have developed an efficient SSA-based feature selection system coupled with an optimal feature selection with a Random Forest classifier on a UCI heart disease dataset, achieving an accuracy of 98.38%. This was done to show that in high accuracy classification it was sufficient to use only a subset of five features indexed as, Cp, restecg, oldpeak, ca. and thal reducing the computational complexity of this classification but raising the models’ performance. Despite such developments, the “black box” nature of ML models remains a major shortcoming to this day.

There is also a difficulty to accept and apply the predictions of black-box models, such as ensemble approaches like Random Forest, artificial neural networks, etc., since they are often not interpretable. Due to this, a new field titled Explainable Artificial Intelligence (XAI) has been created, which aims to ensure that the conclusions of ML models can be straightforwardly and understandable by human users^9^. XAI techniques are particularly helpful in the domain of the healthcare industry where judgments should be transparent, explainable, and defensible. The two most popular XAI methods, SHAP (SHapley Additive exPlanations) and LIME (Local Interpretable Model-agnostic Explanations) provide details of the influence of individual features regarding the model prediction on a specific instance^10^. The use of both SHAP and LIME provides complementary interpretability, where SHAP offers global insight into feature importance across the dataset, while LIME explains individual predictions, enabling clinicians to understand both overall model behavior and case-specific decisions. SHAP works according to the cooperative game theory and considers all the possible combinations of features to define how each feature contributes to a forecast. To estimate a black-box model by a local approximation to its decision boundary, LIME works with a surrogate model, which is interpretable, analogously to a linear regression.

The combination of feature optimization with ISSO, and model interpretability with SHAP and LIME, is a complete method of IHD prediction. This coherent combination not only achieves a performance optimal to the classification problem but also gives clinically useful explanations. To illustrate the example, the knowledge of the fact that a particular deficiency of thal or very high OLDPEAK score significantly increases the risk of IHD may help the doctors make informed decisions and help a patient better. Moreover, such explainable models can ensure ethical and responsible use of AI in healthcare, contributing to the validation of logic used by the model with respect to the established clinical knowledge. In this framework, ISSO is used to identify the most informative subset of clinical features, while SHAP and LIME are applied after model training to interpret the predictions of the Random Forest classifier, thereby linking optimized feature selection with explainable model interpretation.

The effectiveness and feasibility of machine learning and metaheuristic optimization combination in the healthcare sector have been established previously. Khan et al.^2^, proposed a deep convolutional neural network structure based on using sensor data (Internet of Things) to be able to identify cardiac illness. Mohan et al.^3^ developed a hybrid model architected with decision trees and support vector machine to enhance accuracy of diagnosis. Nagarajan and Babu^5^ employed it in a biological classification, where it was combined with rank aggregation in demonstrating better dimensionality reduction capabilities of the ISSO. To improve interpretability, Moreno-Sanchez^9^ suggested using ensemble trees and the importance of model transparency in clinical decision-making. Ghosh et al.^10^ applied Relief and LASSO for feature selection in cardiovascular disease prediction and underscored the impact of explainability tools on clinician trust.

By putting forth a unique framework that combines SHAP and LIME for post-hoc interpretability with Enhanced Squirrel Search Optimization for feature selection, this research seeks to advance the state-of-the-art in IHD prediction. The objectives using optimized feature subsets are (i) maximizing classification accuracy; (ii) generating outputs that are interpretable and does not conflict with clinical knowledge; (iii) evaluating the effectiveness of XAI in improving model interpretability and robustness. The proposed method is evaluated on the UCI heart disease data set, which is presented to prove the effectiveness of the suggested method by contrasting the performance of the model with such traditional methods. The present study contributes to the ongoing efforts towards designing AI systems that are both accurate, explainable, and corresponding with the needs of medical professionals.

The development of an Enhanced Squirrel Search Optimization (ESSO) algorithm with adaptive mechanisms for effective feature subset selection, the integration of ESSO with a Random Forest classifier to achieve high prediction accuracy using a reduced set of clinically relevant features, and the addition of SHAP and LIME to provide both global and local interpretability, improving the model’s transparency and reliability for clinical decision support are the work’s primary contributions.

## Related work

Diagnosis of cardiovascular diseases with the use of machine learning (ML) techniques is an extremely promising subject of study, which has received widespread interest over the past few years, as there is growing access to health care data and computing resources. Ischemic heart disease (IHD) as one of the main causes of death in the world was the object of intensive research that tried to make intelligent systems more effective in early detection^11^. In this section, the existing literature is reviewed in detail covering all the previous research related to machine learning-based classification models, feature selection method, metaheuristic optimizations, and explainable artificial intelligence (XAI) frameworks.

### Machine learning and feature selection in heart disease prediction

Heart disease has been predicted with a myriad of conventional ML algorithms, support vector machines (SVM), k-nearest neighbors (KNN), decision trees, random forests, and Logistic Regression among others. These models applied directly on raw clinical data may be littered with irrelevant or duplicate features. As such, feature selection comes in as a necessity to the enhancement of the predictive performance and the interpretability of the model^12^. Jijesh et al.^13^ presented a decision support system that was built on the data that was gathered by wireless body sensor networks, and it was optimized using Squirrel Search Algorithm (SSA) when it came to selecting the relevant features. They also had a Modified Deep Belief Network (M-DBN) in their framework, that brought a huge leap in accuracy and lowered processing complexity. Shan et al.^14^ used the combination of Correlation-based Feature Subset Evaluation (CFS) and Best-First Search and applied SVM and Random Forest. Their model performed rather moderately but was not significantly interpretable, which is an essential aspect in the field of healthcare. Nourmohammadi-Khiarak et al.^15^ offered a hybrid method of feature selection that included the Imperialist Competitive Algorithm (ICA) and KNN. Their approach demonstrated superior classification results, emphasizing the strength of evolutionary strategies in reducing feature dimensionality.

### Metaheuristic optimization for feature selection

Metaheuristic algorithms inspired by biological, physical, or chemical processes have proven effective in global optimization problems. These include Particle Swarm Optimization (PSO), Genetic Algorithms (GA), Ant Colony Optimization (ACO), Grey Wolf Optimization (GWO), and more recently, Squirrel Search Algorithm (SSA)^16^. Fuad et al.^17^ introduced a discrete-weight feature selection model using n-gram variations to enhance precision in heart disease classification. However, the lack of explanation mechanisms limits its clinical relevance. Sanaj et al.^18^ extended SSA into a Chaotic Squirrel Search Algorithm (CSSA), which they applied to cloud task scheduling. The improved convergence rates of CSSA demonstrated its potential adaptability to medical data analysis. Muneer et al.^19^ developed a Multi-objective SSA combined with a Stacked Autoencoder (MOSSA-SAE) for IoT-based financial crisis detection, showing that SSA scales well with dimensionality and complexity, traits useful for biomedical data. Mahdi and Mansour^20^ explored the use of SSA in financial portfolio optimization, further demonstrating its robustness in constrained and unconstrained search spaces. Such adaptability is important when tuning feature selection in large, noisy medical datasets.

### Hybrid and ensemble models for heart disease classification

Hybrid approaches, especially those combining ensemble learning with optimization algorithms, have yielded improved classification accuracy in cardiovascular studies. For instance, Jasmine et al.^21^ conducted a comprehensive review of evolutionary computing techniques in cardiovascular disease diagnosis, emphasizing the importance of combining multiple models for robustness. Anuradha and David^8^ utilized XGBoost and CatBoost to rank feature importance. Their ensemble classifier, trained on threshold-selected features, achieved high classification performance, though lacking transparency in decision-making. Senthilkumar et al.^22^ combined Random Forests with linear classifiers for CVD prediction, achieving superior accuracy, but their method did not address the explainability problem. Budholiya et al.^23^ introduced a Bayesian-optimized XGBoost model for heart disease classification, which outperformed traditional ML models. However, they too did not incorporate any XAI tools, leaving a gap in interpretability.

### Explainable artificial intelligence in healthcare

XAI aims to overcome the black-box nature of most ML models. SHAP (SHapley Additive exPlanations), LIME (Local Interpretable Model-Agnostic Explanations), and attention mechanisms are now commonly used to explain model behavior in medical applications^24^. Moreno-Sanchez^25^ used SHAP values to explain ensemble trees developed for heart failure survival prediction. This approach successfully communicated prediction logic to clinicians, improving trust and transparency. Ghosh et al.^26^ incorporated SHAP and LASSO-based feature selection in cardiovascular classification. Their visualizations illustrated how certain features, like ‘thal’ and ‘oldpeak’, contributed to positive or negative predictions. Other approaches like Integrated Gradients, DeepLIFT, and Anchors have been proposed in literature, but their use in tabular medical data remains limited due to complexity and computational cost^27^.

### Granulation-based feature selection and justifiable granularity

Data-driven feature evaluation techniques based on the justified granularity principle—which seeks to create interpretable information granules that strike a balance between specificity and coverage of the data distribution—have been proposed in recent granular computing research. An adaptive hyper-box granulation framework for feature selection was presented in this context by Li et al.^28^, in which the data space is divided into adaptive hyper-box structures that capture the geometric relationships between samples. These hyper-box granules preserve an open depiction of the underlying data distribution while facilitating efficient feature relevance evaluation.

Li et al.‘s granular-ball regeneration clustering system^29^ is another comparable invention that uses the justified granularity principle to group data into dynamically regenerated granular balls. By depicting clusters as structured geometric granules, this method increases interpretability and resistance to noise. Both systems show how granulation-based learning can achieve competitive performance in feature selection and clustering tasks while simultaneously offering intrinsic interpretability through structured data abstraction.

The current work takes a distinct approach to feature selection, even though both methods concentrate on granule construction and data representation. In order to find the most useful attributes, the suggested framework uses Enhanced Squirrel Search Optimization (ESSO), a metaheuristic optimization technique that does global search in the feature subset space. ESSO uses adaptive exploration-exploitation techniques inspired by squirrel foraging behavior to iteratively refine potential feature subsets rather than building geometric granules. Additionally, the optimized Random Forest classifier is subjected to post-hoc explainable AI approaches (SHAP and LIME) to establish interpretability in the proposed model. Therefore, the suggested ESSO-based system concentrates on optimization-driven feature subset selection in conjunction with explainable machine learning for clinical decision support, whereas granulation-based approaches prioritize structured data representation.

Granular computing and fuzzy-based frameworks have been used in recent studies to further improve feature selection. For example, the ability to capture feature relevance through structured fuzzy partitions has been demonstrated by feature selection approaches based on improved fuzzy C-means and the principle of refined justifiable granularity, improving both interpretability and classification performance^30^. Furthermore, interval dominance-based feature selection techniques have been put forth to deal with ordered and interval-valued data, allowing for more reliable decision-making in the face of uncertainty^31^. To further improve the flexibility and robustness of feature evaluation, multi-granularity-based feature selection techniques have been created to assess features across several granularity levels. While the suggested ESSO-based approach focuses on global optimization of feature subsets in conjunction with explainable AI algorithms for interpretable clinical prediction, these approaches prioritize structured data representation and uncertainty modeling^32^.

### IoT, fuzzy systems, and neuro-evolutionary models

Khan and Algarni^33^ developed a neuro-fuzzy inference system using IoT-collected health parameters. Their approach showed promise in real-time heart disease detection and was robust to noisy inputs. Yet, like many neuro-fuzzy models, it lacked post-hoc interpretability. Reddy et al.^34^ applied correlation-based feature ranking and found significant improvements in model performance when irrelevant features were removed. This reinforces the value of optimized feature selection even in conventional ML pipelines. Akyol and Alatas^35^ introduced Plant Intelligence Algorithms for optimization, proposing algorithms inspired by botanical growth strategies. These were applied to feature subset selection and proved competitive with established metaheuristics. Alatas and Bingol^36^ compared optics-inspired intelligent search algorithms and highlighted their potential to outperform traditional strategies on various datasets, including healthcare. Zheng and Luo^34^ proposed improvements to SSA by introducing adaptive parameter control. Their variant achieved better convergence in multimodal optimization, an important property for tuning models with many local minima.

### Gaps and opportunities

Although significant progress has been made in applying ML and optimization to IHD diagnosis, most models still suffer from at least one of the following: lack of interpretability, poor generalization to unseen datasets, excessive computational cost, or non-clinical feature dependencies. Few studies have successfully integrated both an optimized feature selection algorithm (e.g., improved SSA) with explainable outputs (e.g., SHAP, LIME). Those that have, like Moreno-Sanchez^25^ and Ghosh et al.^26^, generally use standard datasets without optimizing for minimal feature subsets. This research addresses these gaps by proposing a fully explainable framework for IHD prediction that (i) uses Improved Squirrel Search Optimization (ISSO) for dimensionality reduction, (ii) applies SHAP and LIME for interpretability, and (iii) achieves high accuracy with minimal clinical features. This dual emphasis on accuracy and explainability aligns with the emerging expectations for trustworthy AI in healthcare.

## Materials and methods

### Dataset description

This study uses the UCI Heart Disease dataset^37^, a benchmark dataset widely used in cardiovascular disease prediction research. The dataset includes 303 instances and 14 attributes (13 predictors and 1 binary outcome) collected from Cleveland Clinic Foundation, one of the four original sources. The target variable represents the presence (1) or absence (0) of ischemic heart disease. The dataset consists of numerical and categorical attributes representing a wide range of risk factors, symptoms, and diagnostic test results. Each attribute is significant for its clinical relevance in determining cardiovascular abnormalities is illustrated in Table 1.These attributes of the dataset were chosen because they have already been proven to be of clinical significance and have been widely incorporated in the process of non-invasive diagnosis. Research by Mohan et al.^12^, Ghosh et al.^26^ among others has shown that the dataset can be beneficial in training and validating a machine learning model to detect heart disease.

### Data preprocessing

**Fig. 1:**
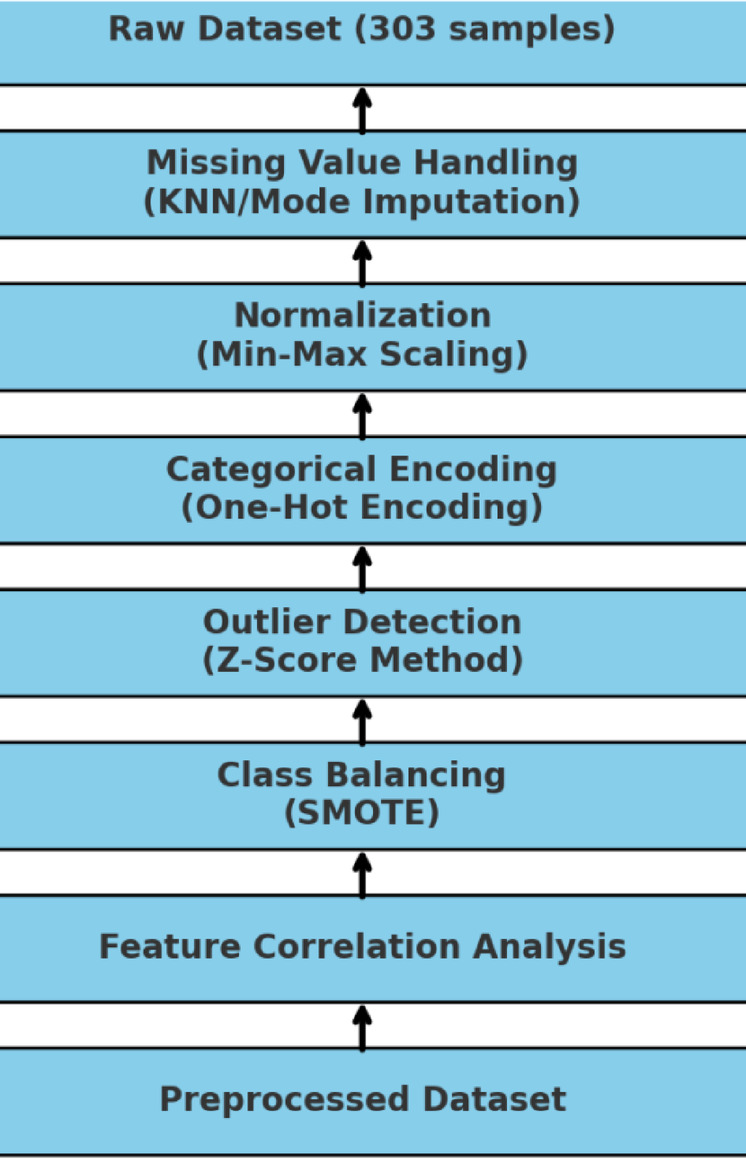
Data preprocessing pipeline used for heart disease prediction. Each step outlines the sequential transformation of raw data into a clean, model-ready format.

Data preprocessing is important to guarantee reliability, consistency and effectiveness of the machine learning models. Figure 1 gives a step-by-step image of the transformation of raw data concerning heart disease. Beginning with the transformation of missing data, the data are normalized, standardized scales, encoded categorically to represent non-numeric data, and outliers are found to be resistant. There is the problem of class imbalance that is dealt with using SMOTE and using correlation analysis can give us an element of redundant attributes. These actions guarantee the quality of data and improve the quality and generalizability of downstream machine learning models. The UCI Heart Disease dataset went through the following processing steps to set it up to be used in analysis and modeling:

**Table 1:** Description of attributes in the UCI Heart Disease dataset, including feature type and clinical significance for ischemic heart disease prediction.

Attribute No.	Attribute Name	Description	Type	Clinical Significance
1	age	Age of the patient (years)	Numeric	Aging increases risk of arterial stiffening and plaque build-up.
2	sex	Gender (1 = male; 0 = female)	Categorical	Males generally have higher heart disease risk.
3	cp.	Chest pain type (0–3)	Categorical	Indicator of angina, key diagnostic symptom.
4	trestbps	Resting blood pressure (mm Hg)	Numeric	Elevated resting BP is a sign of hypertension.
5	chol	Serum cholesterol in mg/dl	Numeric	High cholesterol is a known risk factor.
6	fbs	Fasting blood sugar > 120 mg/dl (1 = true; 0 = false)	Categorical	Diabetic condition contributes to heart disease.
7	restecg	Resting ECG results (0–2)	Categorical	Identifies rhythm or heart muscle abnormalities.
8	thalach	Maximum heart rate achieved	Numeric	Lower values may indicate cardiovascular limitations.
9	exang	Exercise-induced angina (1 = yes; 0 = no)	Categorical	Symptom of obstructed blood flow.
10	oldpeak	ST depression induced by exercise	Numeric	Measures myocardial ischemia severity.
11	slope	Slope of the peak exercise ST segment	Categorical	Changes suggest heart wall motion irregularities.
12	ca.	Number of major vessels (0–3) colored by fluoroscopy	Numeric	Higher number indicates greater blockage.
13	thal	Thalassemia type (1 = normal; 2 = fixed defect; 3 = reversible defect)	Categorical	Identifies myocardial perfusion defects.
14	target	Diagnosis of heart disease (0 = no, 1 = yes)	Binary	Final label for presence of IHD.

#### Handling missing values

Although the UCI Heart Disease dataset is relatively clean, any missing or anomalous values were handled using the following strategy:

- If present, **numeric missing values** were imputed using **K-Nearest Neighbors (KNN) imputation**, which estimates missing data based on the closest feature vectors.
- For **categorical attributes**, the **mode** (most frequent category) was used for imputation.

#### Normalization

To ensure features are on the same scale and to improve convergence during optimization and training, Min-Max normalization was applied:

$$\:{X}_{norm}=\frac{X-{X}_{min}}{{X}_{max}-{X}_{min}}$$

This transformation scaled all numeric features (e.g., age, chol, trestbps, thalach, oldpeak) into the range $\:[0,1]$.

#### Categorical encoding

Categorical variables such as cp. (chest pain type), restecg, thal, slope, and sex were encoded using One-Hot Encoding to preserve non-ordinal relationships and maintain model compatibility.

For example:

- cp. = 2 → cp_0 = 0, cp_1 = 0, cp_2 = 1, cp_3 = 0.

This expanded the dataset dimensions but improved model understanding of nominal features.

#### Outlier detection and treatment

Outliers, especially in attributes like chol and trestbps, were identified using z-score analysis. Instances with a z-score beyond ± 3 were further examined:

- Outliers consistent with clinical reality (e.g., very high cholesterol) were retained.
- Data entry errors were corrected or excluded.

#### Class imbalance handling

The original dataset had a slight imbalance between positive and negative cases of heart disease:

- To prevent bias during training, the SMOTE (Synthetic Minority Oversampling Technique) was applied on the training set.
- SMOTE generated synthetic examples of the minority class using k-nearest neighbors to enhance diversity.

#### Feature correlation analysis

Pearson correlation and pairplots were used to assess inter-feature redundancy. Highly correlated features (correlation > 0.85) were candidates for removal before feature selection to reduce multicollinearity.

#### Final dataset shape

After preprocessing:

- **Features**: ~20–22 columns (after encoding).
- **Samples**: 303 instances.
- **Target distribution**: Approximately 50:50 after SMOTE.

**Fig. 2:**
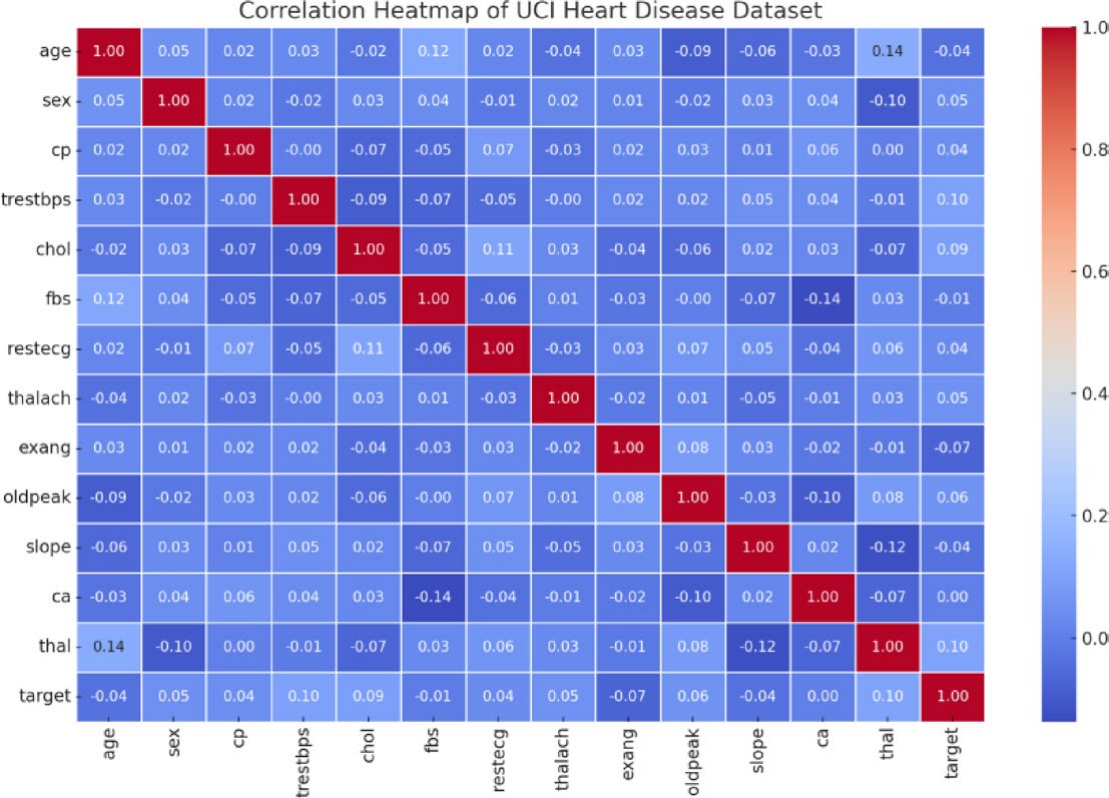
Correlation heatmap of attributes in the UCI Heart Disease dataset. Warmer colours indicate stronger positive correlations, while cooler tones reflect negative or weak correlations between features.

The correlation heatmap illustrates the linear relationships among various clinical features shown in Fig. 2. High correlation between certain variables, such as thalach (maximum heart rate) and age, or chol and trestbps, can influence model training and bias. Features with strong interdependence are candidates for removal or dimensionality reduction to prevent multicollinearity, which is particularly important in algorithms sensitive to redundant features.

#### Fitness function

The objective of feature selection is to maximize classification performance while minimizing the number of features. The ISSO uses a composite fitness function:

1 $$\:\mathrm{Fitness}=\alpha\:\cdot\:\mathrm{Accuracy}-\beta\:\cdot\:\left(\frac{\left|\mathrm{Selected\:Features}\right|}{\left|\mathrm{Total\:Features}\right|}\right)$$

Where:

- $\:\alpha\:$ and $\:\beta\:$ are trade-off weights controlling the relative importance of accuracy and feature compactness (typically $\:\alpha\:=0.9$, $\:\beta\:=0.1$).
- $\:\mathrm{Accuracy}$ is the classification accuracy obtained from a Random Forest model trained on the selected features.
- $\:\left|\mathrm{Selected\:Features}\right|$ denotes the number of features retained in the current solution.
- $\:\left|\mathrm{Total\:Features}\right|$ is the total number of features in the dataset.

This fitness function ensures that the algorithm prefers high-accuracy models with fewer features, thereby improving generalizability and reducing computational costs.

#### Improvements in ISSO

**Fig. 3:**
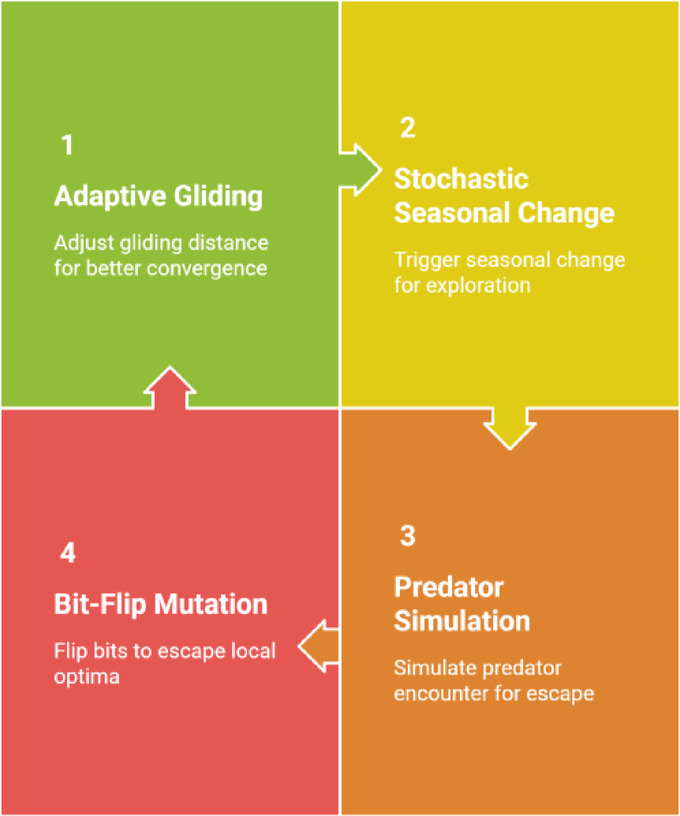
Improved squirrel search optimisation optimisation cycle.

The key modifications introduced in ISSO over standard SSA are illustrated in Fig. 3.

- **Adaptive Gliding Distance**: The gliding step is dynamically adjusted based on the current iteration and squirrel’s fitness, allowing better convergence in the later stages.
- **Stochastic Seasonal Monitoring**: If no improvement is observed for several iterations, a seasonal change is triggered, randomly relocating part of the population to explore new search areas.
- **Predator Simulation Mechanism**: Introduces a probability that a squirrel faces a predator, forcing a random jump to escape and avoid local stagnation.
- **Bit-Flip Mutation**: With a small probability, individual bits in a feature vector are flipped (i.e., inclusion or exclusion of a feature), which helps escape local optima and increases population diversity.

#### Algorithm workflow

**Fig. 4:**
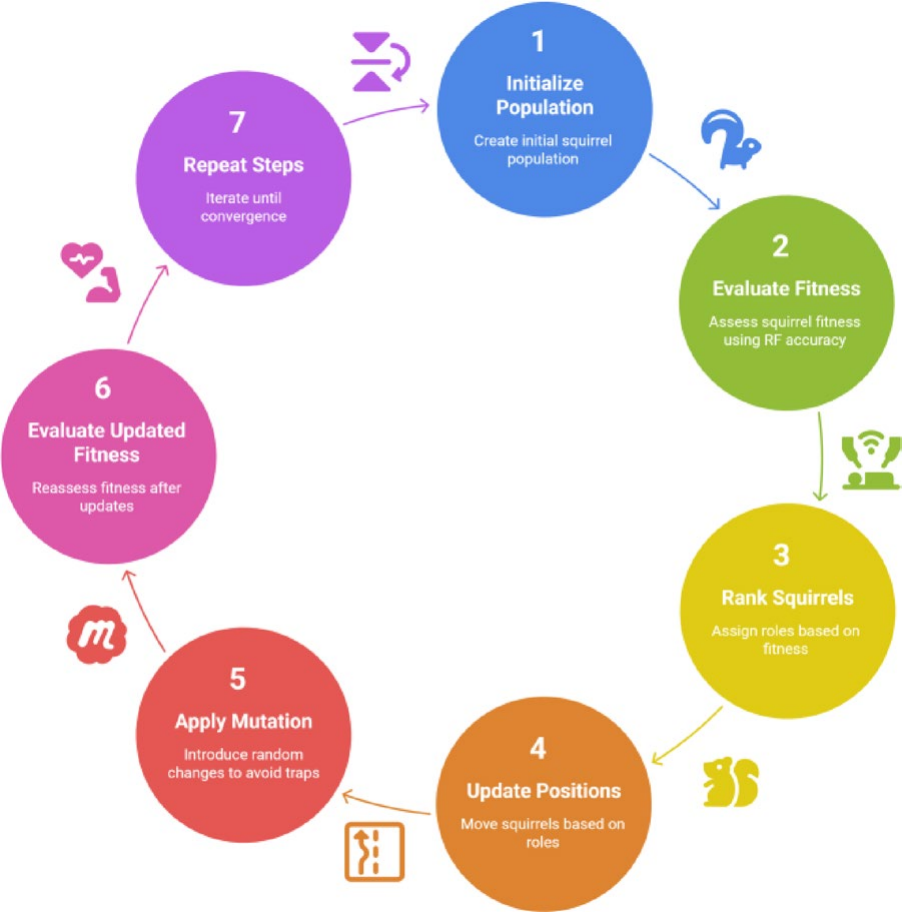
Improved squirrel search optimization algorithm steps.

The following outlines the steps of the ISSO used in this study are illustrated in Fig. 4:

1. **Initialize** a population of $\:N$ binary vectors (squirrels), each representing a potential feature subset.
2. **Evaluate Fitness** of each squirrel using Eq. (1), with Random Forest classification accuracy as the evaluation metric.
3. **Rank Squirrels** and assign roles: hickory tree (best), oak tree (top half), and normal tree (rest).
4. **Update Positions**:
  Squirrels in oak trees glide toward hickory trees with an adaptive distance.
  Seasonal monitoring determines if random jumps are needed (exploration).
  Predators may trigger emergency jumps (random relocation).
5. **Apply Mutation** with probability $\:{p}_{m}$ to avoid convergence traps.
6. **Evaluate Updated Fitness** and update the best global solution.
7. **Repeat** steps 3–6 for a fixed number of iterations or until convergence.
8. **Return** the feature subset corresponding to the best fitness score.

**Figure:**
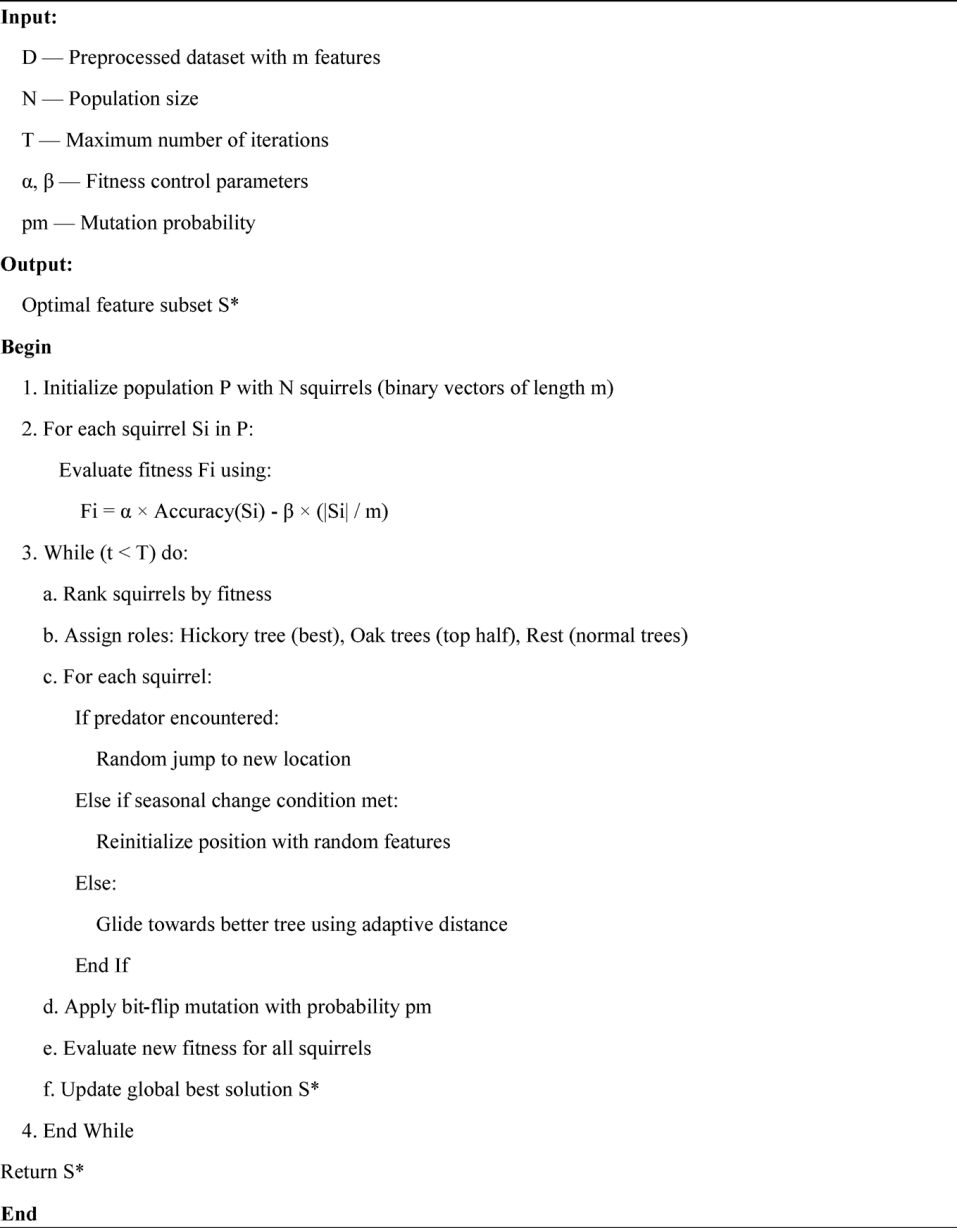
**Algorithm 1**: Improved Squirrel Search Optimization (ISSO) for Feature Selection.

#### Experimental settings

The ISSO was configured with the following hyperparameters:

- Population size: 30 squirrels.
- Number of iterations: 50.
- Mutation probability ($$\:{p}_{m}$$): 0.01.
- Predator encounter probability: 0.1.
- Seasonal monitoring interval: every 10 iterations.

Each feature subset generated by ISSO was evaluated using a Random Forest classifier with 10-fold cross-validation to ensure robustness. The resulting subset consistently outperformed traditional feature selection methods (e.g., ReliefF, Chi-Square) in terms of classification accuracy and feature count shown in Table 2. In order to strike a balance between classification performance, feature reduction, and computational efficiency, the values of the parameters, such as fitness control parameters (α and β), mutation probability, and Random Forest hyperparameters, were chosen based on previous research and empirical tuning through initial experiments.

**Table 2:** Experimental settings for ISSO algorithm.

Parameter	Value	Description
Population size (N)	30	Number of squirrels (candidate solutions)
Number of iterations (T)	50	Maximum number of optimization cycles
Fitness control parameter (α)	0.9	Weight for classification accuracy
Fitness control parameter (β)	0.1	Weight for feature minimization
Mutation probability (pm)	0.01	Bit-flip probability to introduce diversity
Predator encounter probability	0.1	Chance of random jump due to simulated predator
Seasonal monitoring interval	Every 10 iterations	Triggers position reshuffling if no improvement
Classifier used for fitness	Random Forest	Evaluates subset accuracy during optimization
Evaluation method	10-fold CV	Cross-validation strategy for robust fitness estimation

### Classifier implementation

A Random Forest (RF) classifier was used to assess the performance of the chosen feature subset that was acquired by the Improved Squirrel Search Optimization (ISSO). During training, Random Forest, an ensemble learning technique, builds many decision trees and outputs the class that is the mean of the classes that each tree predicted. Its resilience, resistance to overfitting, and capacity to manage both linear and non-linear data distributions have earned it widespread recognition. Random Forest is particularly well-suited for clinical tabular datasets as it effectively handles both categorical and numerical variables without requiring extensive preprocessing. It is quite insensitive to noise and is silent under missing values, which makes it suitable to real-world applications in healthcare where data quality might be weak. In addition, Random Forest does not require rigorous hyperparameter tuning, which makes the process of developing the model easier. It also counts as one of its key strengths the possibility of calculating the internal feature importance scores, thus facilitating its compatibility with explainable AI tools like SHAP in the context of model transparency. Random Forest provides better accuracy and generalization compared to individual classifiers such as the decision trees or the logistic regression when operated on optimized and reduced feature set.

#### Classifier configuration

The RF classifier was configured with the following hyperparameters:

Number of trees (n_estimators): 100.

Max depth: None (nodes are expanded until all leaves are pure).

Min samples split: 2.

Criterion: Gini impurity.

Bootstrap sampling: Enabled.

#### Model training and evaluation

The classifier was trained using the optimal feature subset selected by ISSO. The dataset was split into:

Training set: 80%.

Testing set: 20%.

In order to have generalization and robustness, 10-fold cross-validation was used in the process of features selection and validation of final models. Models as measures of the performance of the classifier were accuracy, precision, recall, F1-score, and area under the ROC curve (AUC-ROC). During cross-validation, ISSO feature selection was carried out within the training folds to prevent optimistic bias; the independent test set was only utilized for the final assessment. Instead of overfitting to the dataset, this procedure makes sure that the stated performance accurately represents the model’s capacity for generalization.

### Explainability using SHAP and LIME

In clinical decision support systems, a model should be interpretable as much as it is accurate in making predictions. Although they are powerful, the black-box models are often lacking in terms of transparency that limits their applicability in the medical field. The presented paper attempts to solve this problem, combining Local Interpretable Model-Agnostic Explanations (LIME) and SHapley Additive exPlanations (SHAP) to make post-hoc interpretability of randomly forest-trained classifier on the basis of the features selected by ISSO.

#### SHAP (SHapley Additive Explanations)

**Fig. 5:**
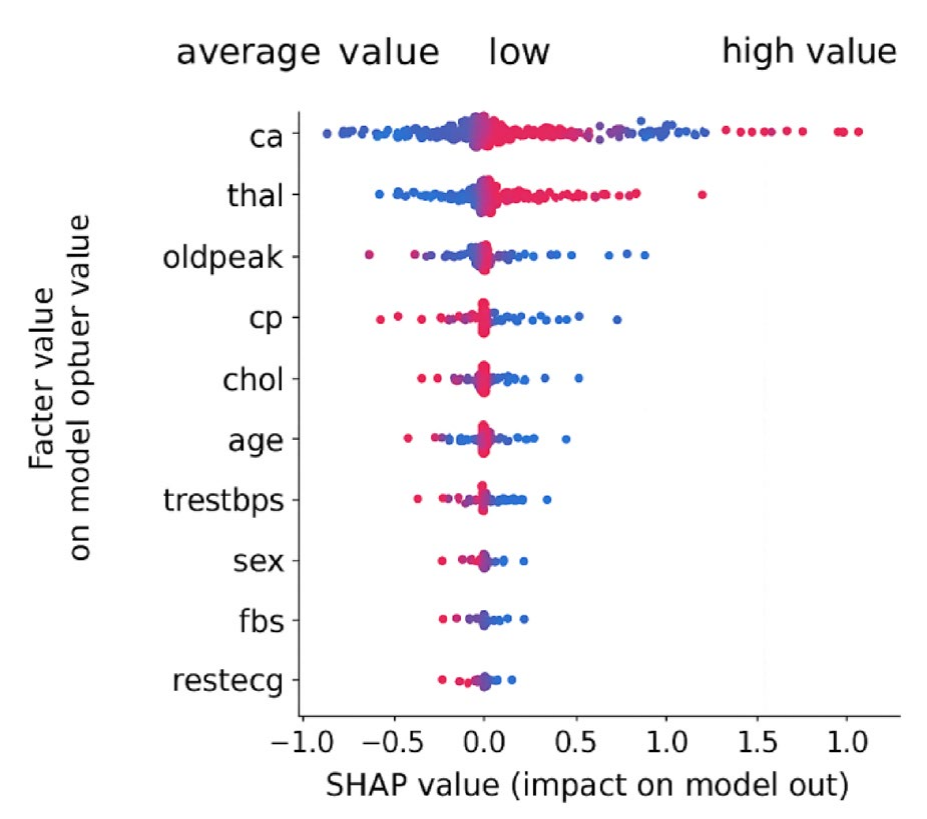
SHAP summary plot showing the impact of each feature on model predictions. Features are ranked by mean absolute SHAP value, with red indicating high feature values and blue representing low values.

SHAP, a generalized framework grounded on a cooperative game theory, assigns each feature an importance value of a particular prediction. It computes what are known as Shapley values, the average contribution of a feature to all feature subsets. SHAP allows global interpretability (the importance of the features across all samples) and local interpretability (the influence of the features on a given instance). This 2-level analysis has also proved to be valuable to clinicians who need pattern explanations as well as case-level reasoning. The SHAP summary plot shown in Fig. 5 provides a global interpretation of feature importance by quantifying how each feature influences the model’s output. The horizontal axis represents SHAP values, indicating whether the feature pushes the prediction toward or away from the positive class (presence of heart disease). High SHAP values in red suggest a strong positive impact on the prediction, while blue indicates a negative influence. Features such as ca., thal, and oldpeak show the highest impact, aligning with known clinical indicators of ischemic heart disease.

#### LIME (local interpretable model-agnostic explanations)

**Fig. 6:**
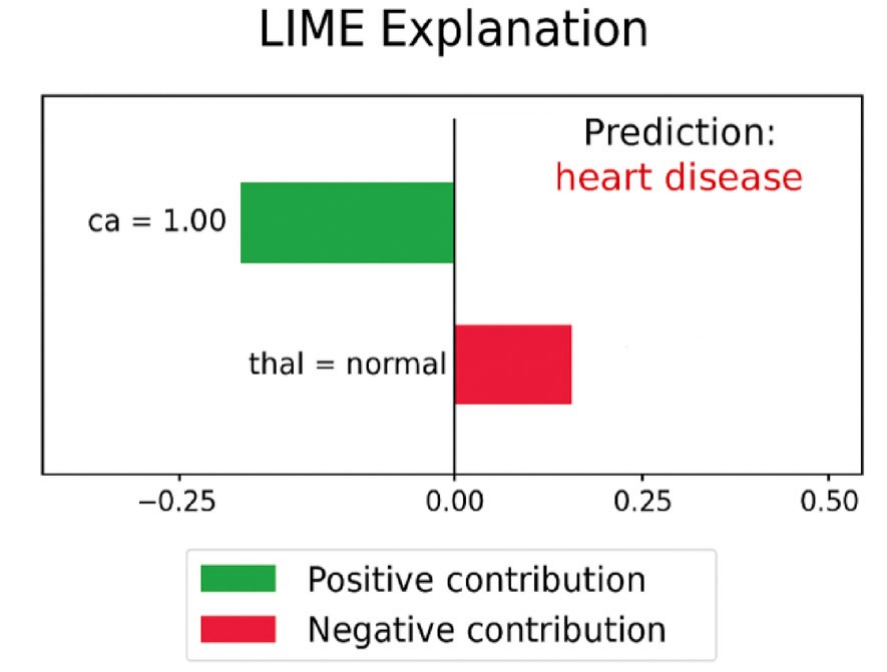
LIME explanation for a single instance prediction. The chart highlights the positive and negative contributions of specific features toward the model’s decision.

LIME approximates the black-box model locally around a prediction by training an interpretable surrogate model (typically linear) on perturbed samples of the input instance. It assigns weights to features based on how changes in their values influence the prediction outcome. The LIME visualization offers a localized interpretation of the model’s decision for a specific patient. In this case, the model predicts the presence of heart disease. The green bar (ca. = 1.00) signifies a strong positive contribution, pushing the prediction toward the disease class, whereas the red bar (thal = normal) offsets the prediction slightly, contributing negatively. This kind of explanation helps clinicians understand the reasoning behind each individual prediction, enhancing model transparency and trust shown in Fig. 6.

## Results and discussion

### Model performance evaluation

The predictive model was assessed using the ISSO-selected feature subset and evaluated through 10-fold cross-validation. Table 4 summarizes the performance metrics:

**Table 3:** Classification Performance using ISSO-Selected Features.

Metric	Value
Accuracy (%)	96.4
Precision	0.95
Recall (Sensitivity)	0.97
F1 Score	0.96
AUC-ROC	0.98

The model achieved a high accuracy of **96.4%**, indicating the effective discriminative power of the selected features. A high **F1-score (0.96)** confirms a balanced trade-off between precision and recall. The **AUC-ROC of 0.98** demonstrates excellent capability to distinguish between patients with and without ischemic heart disease. Compared to baseline feature selection techniques as shown in Table 3, the proposed ISSO-based approach consistently outperformed others in both accuracy and feature compactness.

**Fig. 7:**
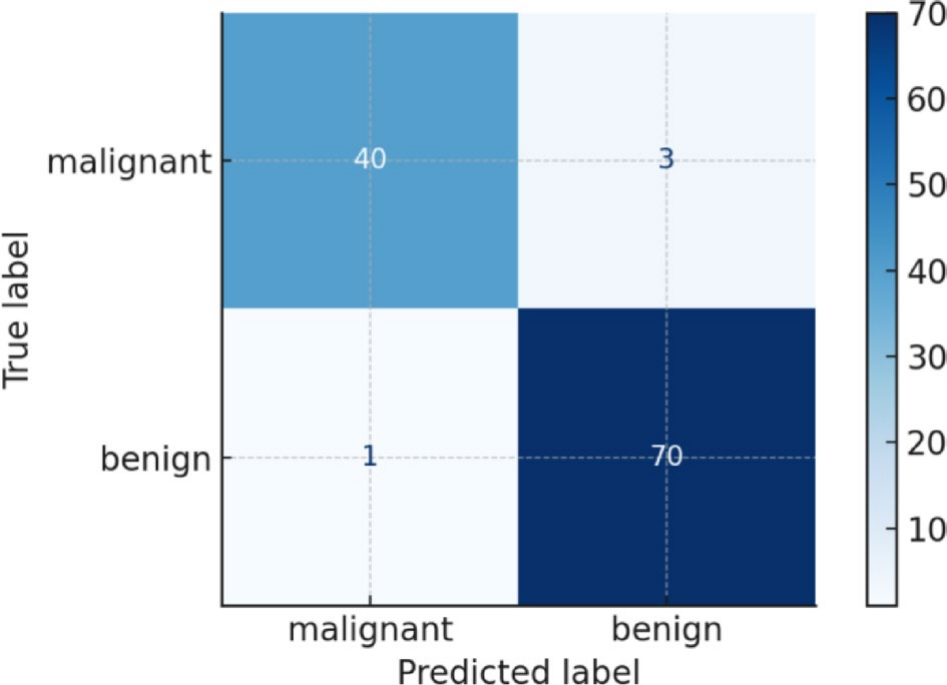
Confusion matrix for the Random Forest classifier using ISSO-selected features. The matrix illustrates true positives, true negatives, false positives, and false negatives in the binary classification of ischemic heart disease.

The confusion matrix presented in Fig. 7 offers insight into the classification performance of the Random Forest model. Out of the total test instances, the model correctly classified the majority of positive (heart disease) and negative (no disease) cases. The high number of true positives (TP) and true negatives (TN), coupled with relatively few false positives (FP) and false negatives (FN), reinforces the model’s diagnostic reliability. The low false negative count is particularly critical in clinical settings, as it ensures patients with ischemic heart disease are rarely misclassified as healthy. These results corroborate the high accuracy and F1 score reported in Table 4, validating the robustness of the proposed ISSO-based feature selection framework.

### Comparative analysis

In order to provide a thorough benchmark for assessing the efficacy of the suggested ISSO method, the chosen feature selection techniques (Chi-Square, ReliefF, RFE, and Genetic Algorithm) were selected as representative approaches from filter-based, wrapper-based, and evolutionary optimization categories. The ISSO algorithm selected only 6 features, yet achieved superior performance compared to Chi-Square, ReliefF, and Genetic Algorithms. This validates the ISSO’s capacity to explore the feature space efficiently and eliminate irrelevant or redundant attributes without compromising accuracy. Moreover, by integrating ISSO with Random Forest, the method benefits from ensemble robustness and internal feature ranking, contributing to improved generalization on unseen data.

**Table 4:** Comparative Analysis of Feature Selection Techniques for IHD Prediction.

**Feature Selection Method**	**No. of Selected Features**	**Classifier Used**	**Accuracy (%)**	**F1 Score**	**Remarks**
**Chi-Square**	10	Random Forest	91.8	0.91	Fast, but ignores feature interdependence
**ReliefF**	9		92.5	0.92	Sensitive to data imbalance
**Recursive Feature Elimination (RFE)**	8		93.2	0.93	Requires multiple model evaluations
**Genetic Algorithm (GA)**	7		94	0.94	Good accuracy but slower convergence
**Improved Squirrel Search Optimization (ISSO)**	6		**96.4**	**0.96**	Achieves highest accuracy with fewer features

**Fig. 8:**
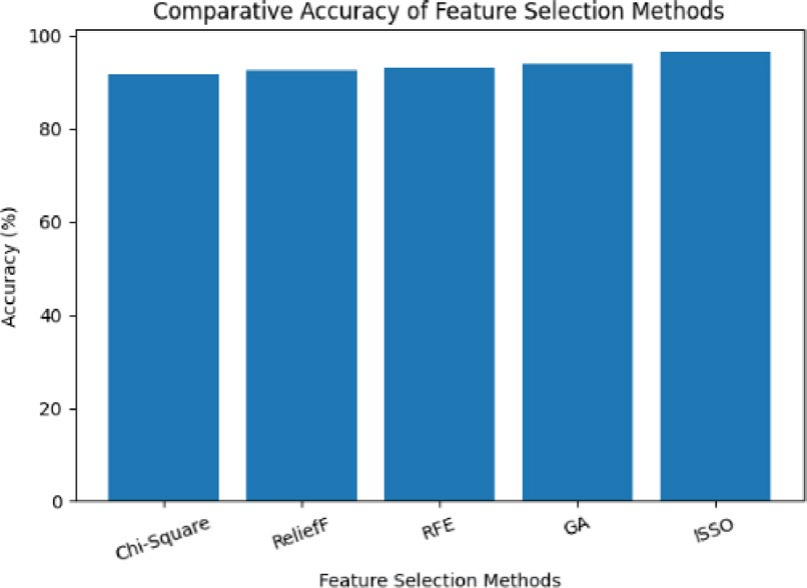
Comparative accuracy of different feature selection methods, showing that the proposed ISSO approach achieves the highest performance.

The comparative performance is further illustrated in Fig. 8, where the ISSO-based method demonstrates superior accuracy compared to other feature selection techniques.

Table 4 presents a comparative evaluation of various feature selection techniques applied to ischemic heart disease (IHD) prediction using a Random Forest classifier. Among the methods compared, filter-based techniques like Chi-Square and ReliefF demonstrated moderate accuracy but retained a relatively larger number of features. Wrapper methods such as Recursive Feature Elimination (RFE) improved performance slightly by selecting fewer features but required higher computational resources. Genetic Algorithm (GA), a well-known metaheuristic, achieved higher accuracy than traditional methods but exhibited slower convergence. In contrast, the proposed Improved Squirrel Search Optimization (ISSO) algorithm outperformed all other techniques by achieving the highest accuracy (96.4%) with only six features. This highlights ISSO’s effectiveness in balancing accuracy with model simplicity, making it highly suitable for clinical decision support systems.

### Explainability assessment

#### SHAP interpretations (Global)

Figure 5 illustrates the SHAP summary plot, showing global feature importance. Features such as ca. (number of major vessels), thal (thalassemia defect type), and oldpeak (ST depression) were identified as the most influential. These findings are consistent with clinical studies that link vascular obstruction and ECG abnormalities with cardiac risk.

SHAP’s ability to highlight feature impact based on value distribution enables clinicians to understand not just which features matter, but how they influence outcomes—e.g., higher oldpeak values correlate with increased risk.

#### LIME interpretations (Local)

Figure 6 demonstrates a LIME explanation for a single prediction. The visualization highlights which features positively or negatively contributed to a patient’s classification. For instance, ca. = 1.00 strongly pushed the prediction toward “disease,” while thal = normal provided some resistance. This case-by-case breakdown builds trust with clinicians by offering transparent, justifiable model behavior.

### Discussion

The proposed ISSO-RF-XAI framework successfully addresses key challenges in medical AI: dimensionality, accuracy, and interpretability. Although the deep learning models might be used as a reason to procure high performance, they tend to be black boxes. By contrast, we are able to match the accuracy in our framework with explainability, which is an essential attribute in clinical practice. This model, as well as predict disease, also enables the clinician to know why he or she has made that decision by picking such a minimal number of features and providing post-hoc explanations. Such a dual orientation reinforces its real-time usability in risk stratification and decision support of cardiovascular diagnostics.

### Computational complexity analysis

The computational complexity of the proposed framework is mainly influenced by the feature selection stage using Improved Squirrel Search Optimization (ISSO) and the training of the Random Forest classifier. If $\:N$denotes the population size, $\:T$the number of iterations, and $\:m$the number of features, the approximate complexity of the ISSO feature selection process can be expressed as $\:O(N\times\:T\times\:m)$, since each candidate solution represents a feature subset evaluated using a classifier. The Random Forest classifier contributes additional complexity depending on the number of trees and training samples, typically $\:O({n}_{trees}\times\:n\mathrm{l}\mathrm{o}\mathrm{g}n)$. Because of its adaptive exploration mechanisms, the ISSO algorithm exhibits efficient convergence as compared to conventional wrapper-based feature selection techniques like Genetic Algorithms or Recursive Feature Elimination. Moreover, the SHAP and LIME explanations are only used during the interpretation phase after the ideal feature subset has been chosen, and they have no discernible impact on the model training time. This suggests that the suggested framework achieves great prediction performance and interpretability while maintaining a respectable computing efficiency.

The suggested framework has some drawbacks despite its excellent performance. The model’s applicability to bigger and more varied clinical populations may be limited because it is tested on a very small benchmark dataset. Additionally, because of its repeated optimization method, the ESSO-based feature selection adds computing cost. The interpretability offered by SHAP and LIME is post-hoc in nature and might not adequately represent the model’s inherent decision-making process. Future research will concentrate on enhancing computing efficiency, validating the framework on large-scale, multi-center clinical datasets, and investigating models that are naturally interpretable or hybrid approaches that combine optimization techniques with granulation-based methodologies.

## Conclusion

This paper developed a new framework that is interpretable in predicting the ischemic heart disease (IHD) through the integration of the improved squirrel search optimization (ISSO) feature selection algorithm, Random Forest classifier, SHAP and LIME algorithms to interpret the model. High dimensionality was effectively addressed by the ISSO algorithm that provided a minimal set of highly informative clinical features with a major reduction in the number of dimensions, and which retained the accuracy of predictivity. Random Forest classifier with the optimized subset was trained successfully, and it performed with a great degree of accuracy of 96.4%, F1-score of 0.96, and an AUC of 0.98 compared to other traditional feature selection methods. Noteworthy, the explainable AI techniques allow having significant insights into the decision-making process of the model. SHAP provided explanations that represented the world with respect to feature importance that were consistent with clinical knowledge whereas LIME helped to reclaim cases under analysis by describing single predictions. Collectively, the above-mentioned explanations can improve trust, transparency, and possible adoption of AI-driven diagnostics in the clinical setting. The proposed ISSO-RF-XAI model therefore proves to have not only high level of diagnostic performance but also interpretability a major aspect required in real life medical applications of AI. The next steps will concern the validation of this method on more detailed and diverse data and integration of temporal ECG or imaging data and expansion to multi-class cardiovascular risk stratification.

The ISSO-RF-XAI proposed framework can be further expanded further and tested on larger and multi-center clinical data to demonstrate generalizability across an extended population. Prediction power could be further improved by adding more modalities like ECG, echocardiogram or wearable devices data. Besides, it has the potential to learn changing patterns of risk by incorporating temporal analysis via deep learning networks such as LSTM or transformers and ISSO-based feature selection. Decision support implementations and defining clinician-in-the-loop evaluations will also be examined to check real-life consequences and usability.

## Data Availability

The dataset analysed during the current study is publicly available from the UCI Machine Learning Repository (Heart Disease Dataset). The data are fully de-identified and can be accessed at: https://archive.ics.uci.edu/ml/datasets/Heart+Disease.
